# Soluble vascular endothelial growth factor receptor-3 suppresses lymphangiogenesis and lymphatic metastasis in bladder cancer

**DOI:** 10.1186/1476-4598-10-36

**Published:** 2011-04-11

**Authors:** Hanseul Yang, Chan Kim, Min-Ju Kim, Reto A Schwendener, Kari Alitalo, Warren Heston, Injune Kim, Wun-Jae Kim, Gou Young Koh

**Affiliations:** 1 National Research Laboratory of Vascular Biology and Graduate School of Medical Science and Engineering, Korea Advanced Institute of Science and Technology (KAIST), Daejeon, 305-701, Republic of Korea; 2 Department of Urology, College of Medicine, Institute for Tumor Research, Chungbuk National University, Cheongju, 360-711, Republic of Korea; 3 Laboratory of Liposome Research, Institute of Molecular Cancer Research, University of Zurich, Zurich, Switzerland; 4 The Molecular/Cancer Biology Laboratory, Biomedicum Helsinki, University of Helsinki, Helsinki, Finland; 5 Department of Cancer Biology, The Lerner Research Institute, The Cleveland Clinic Foundation, Cleveland, OH, USA

## Abstract

**Background:**

Most bladder cancer patients experience lymphatic metastasis in the course of disease progression, yet the relationship between lymphangiogenesis and lymphatic metastasis is not well known. The aim of this study is to elucidate underlying mechanisms of how expanded lymphatic vessels and tumor microenvironment interacts each other and to find effective therapeutic options to inhibit lymphatic metastasis.

**Results:**

The orthotopic urinary bladder cancer (OUBC) model was generated by intravesical injection of MBT-2 cell lines. We investigated the angiogenesis, lymphangiogenesis, and CD11b^+^/CD68^+ ^tumor-associated macrophages (TAM) by using immunofluorescence staining. OUBC displayed a profound lymphangiogenesis and massive infiltration of TAM in primary tumor and lymphatic metastasis in lymph nodes. TAM flocked near lymphatic vessels and express higher levels of VEGF-C/D than CD11b^- ^cells. Because VEGFR-3 was highly expressed in lymphatic vascular endothelial cells, TAM could assist lymphangiogenesis by paracrine manner in bladder tumor. VEGFR-3 expressing adenovirus was administered to block VEGF-C/D signaling pathway and clodronate liposome was used to deplete TAM. The blockade of VEGF-C/D with soluble VEGF receptor-3 markedly inhibited lymphangiogenesis and lymphatic metastasis in OUBC. In addition, the depletion of TAM with clodronate liposome exerted similar effects on OUBC.

**Conclusion:**

VEGF-C/D are the main factors of lymphangiogenesis and lymphatic metastasis in bladder cancer. Moreover, TAM plays an important role in these processes by producing VEGF-C/D. The inhibition of lymphangiogenesis could provide another therapeutic target to inhibit lymphatic metastasis and recurrence in patients with invasive bladder cancer.

## Background

Bladder cancer ranks as the second most common genitourinary cancer and is associated with frequent distant metastasis at the time of both initial diagnosis and recurrence [1,2]. Even though radical cystectomy and lymph node dissection may be curative, about 50% of patients with muscle-invasive bladder cancer eventually experience recurrence and metastases within 2 years of surgery, and most of them die of the disease [1]. In patients with bladder cancer, the presence of metastasis in regional lymph node is a strong indicator of high recurrence (~55% at 5 years after cystectomy) and relatively poor survival rate (~45% at 5 years after cystectomy) [3,4]. Intriguingly, patients and mice with bladder cancer display profound lymphatic vessels in the peripheral and central regions, which could be actively involved in lymphatic metastasis to lymph node [5,6]. However, the underlying causes and driving forces of the expansion of lymphatic vessels in bladder cancer and underlying mechanisms of how the expanded lymphatic vessels are involved in bladder cancer metastasis to lymph nodes are poorly understood.

New lymphatic vessel formation, lymphangiogenesis, is potently induced by lymphangiogenic growth factors such as vascular endothelial growth factor (VEGF)-C and VEGF-D (VEGF-C/D) through binding and activation of their receptor tyrosine kinase, VEGF receptor-3 (VEGFR-3) [7,8]. Proliferation, sprouting, expansion, enlargement and permeability of lymphatic vessels are largely affected by VEGF-C/D, and these processes facilitate cancer cell migration into the lymphatic vessels and spread to sentinel lymph node (SLN) [9-11]. Accordingly, blocking the VEGF-C/D-VEGFR-3 signaling pathway suppresses tumor lymphangiogenesis and lymph node metastasis in several tumor models [12-15]. These reports indicate that VEGF-C/D-mediated VEGFR-3 activation contributes to not only lymphatic vessel growth but also tumor cell dissemination via lymphatic vessels. In addition, VEGF-C/D is highly expressed in patients with bladder cancer [16,17] and this expression is closely related to lymph node metastasis, but it has an inconsistent relationship with prognosis and survival rate. Yet, causative relationship between expression of VEGF-C/D, lymphangiogenesis and lymph node metastasis in the bladder cancer is not exactly known. It is known that CD11b^+ ^tumor-associated macrophages (TAM) mainly express genes of the M2 type such as CD68, F4/80 and VEGFR-1, and is actively involved in tumor invasion and progression [18]. Accumulating evidences indicate that TAM takes considerable part in promoting tumor angiogenesis and progression by abundant secretions of diverse angiogenic and lymphangiogenic growth factors [18-23]. However, involvement of TAM in lymphangiogenesis and lymphatic metastasis to SLN is unknown in the bladder cancer. Therefore, in this study, we investigated roles of VEGF-C/D and involvement of TAM in tumor lymphangiogenesis and lymphatic metastasis in the murine orthotopic urinary bladder cancer (OUBC) model. We found that OUBC induces profound lymphangiogenesis in the tumor and SLN and substantial lymphatic metastasis to SLN through VEGF-C/D-VEGFR-3 signaling pathway. Furthermore, CD11b^+^/CD68^+ ^TAM was largely infiltrated around the tumor lymphatic vessels, and they were closely involved in the OUBC-induced lymphangiogenesis and lymphatic metastasis, possibly through secretary lymphangiogenic factors such as VEGF-C/D. Our findings provide a novel insight and useful treatment strategy how to control lymphatic metastasis in the patients with bladder cancer through suppression of VEGFR-3 signaling pathway.

## Methods

### Generation of OUBC mouse model

Eight-ten weeks old female C3H/HeN mice were purchased from the Orient Bio Inc. (Seongnam, Korea). Animal care and experimental procedures were performed under approval from the Animal Care Committees of KAIST. The murine bladder cancer cell line MBT-2 was originally obtained from Dr. Timothy Ratliff (University of Iowa, Iowa City, Iowa). The cell line initially originated from a transplantable N-[4-(5-nitro-2-furyl)-2-thiazolyl]-formamide-induced bladder tumor in a C3H mouse [24]. The MBT-2 cells were cultured in DMEM (Lonza, Walkersville, MD) containing 10% heat-inactivated fetal bovine serum (Cambrex, Walkersville, MD), 50 U/ml penicillin and 50 mg/ml streptomycin (Invitrogen, Grand Island, NY) in plastic tissue culture dishes at 37°C in a humidified atmosphere of 5% CO_2_. The cells were trypsinized and resuspended to a single cell suspension in PBS. The mice were anesthetized with an intramuscular injection of ketamine (100 mg/kg) and xylazine (10 mg/kg). A 24G catheter (Becton Dickinson Korea Ltd., Korea) was inserted into the bladder through the urethra. The bladder epithelial layer was wounded with 100 μl of 0.1N HCl and NaOH to improve tumor cell implantation. 1 × 10^6 ^suspended MBT-2 cells in 100 μl of PBS were injected into the bladder cavity.

### Histologic and morphometric analysis

Four weeks after MBT-2 cell implantation, bladder and SLN were sampled. For whole-mount staining, tissue samples were fixed in 1% paraformadehyde in PBS for 1 hr at room temperature (RT). Whole-mounted tissues were incubated for 3 hr at RT with hamster monoclonal anti-platelet endothelial cell adhesion molecule-1 (PECAM-1) antibody 1:1,000 (Chemicon International, Temecula, CA), or rabbit polyclonal anti-lymphatic vessel endothelial hyaluronan receptor-1 (LYVE-1) antibody 1:1000 (Upstate, Lake Placid, NY) after a blocking the fixed tissues with 5% donkey serum in PBST (0.3% Triton X-100 in PBS) for 1 hr at RT. Samples were incubated for 2 hrs at RT with HRP-conjugated anti-hamster antibody 1:100 (Jackson ImmunoResearch, West Grove, PA) or HRP-conjugated anti-rabbit antibody, 1:100 (Amersham, Piscataway, NJ) and developed with 3,3'-diaminobenzidine (DAB) substrate kit according to manufacturer's instruction (Vector, Burlingame, CA). Samples were analyzed and photographed with a Zeiss Stemi SV6 stereomicroscope. For hematoxylin-eosin (H&E) and immunofluorescent staining of tissue sections, Samples were fixed in acetone for overnight at -20°C. Samples were embedded in paraffin block after serial incubation with methyl benzoate and xylene for 30 min at RT. Paraffin blocks were cut in 5-10 μm sections using Microm HM 335E rotary microtome (IMEB Inc, San Marcos, CA). Tissue sections were blocked with 5% goat or donkey serum in TBST (0.03% Triton X-100 in TBS) after dissolving the paraffin with xylene and acetone, immunostained with antibodies for 3 hr; these included hamster monoclonal anti-PECAM-1 antibody 1:200, rabbit polyclonal anti-LYVE-1 antibody 1:200, rat monoclonal anti-CD11b antibody 1:100 (BD Pharmingen, Franklin Lakes, NJ), rat monoclonal FITC-conjugated anti-CD11b antibody 1:50 (BD Pharminogen), mouse monoclonal anti-Cytokeratin antibody (Abcam Inc, Cambridge, MA), rat monoclonal FITC-conjugated anti-CD68 antibody 1:100 (Serotec Ltd., Oxford, UK), rat monoclonal anti-F4/80 antibody 1:100 (Research Diagnostics Inc., Flanders, NJ), goat polyclonal anti-VEGFR-3 antibody 1:100 (R&D Systems, Inc., Minneapolis, MN), goat polyclonal anti-VEGF-C antibody 1:100 (Santa Cruz Biotechnology, Inc.). After several washing steps using TBS, FITC-conjugated anti-hamster IgG antibody 1:1000, FITC-conjugated anti-rat IgG antibody 1:1000, FITC-conjugated anti-mouse IgG antibody 1:1000, Cy3-conjugated anti-rabbit IgG antibody 1:1000, Cy3-conjugated anti-rat IgG antibody 1:1000, Rhodamine-conjugated anti-goat IgG antibody 1:1000 were used as secondary antibodies. All secondary antibodies were purchased from Jackson ImmunoResearch Laboratories, Inc. Goat Fab fragment anti-mIgG (Jackson ImmunoResearch Laboratories, Inc.) was used to block endogenous mouse IgG to use mouse antibody on mouse tissue. Fluorescent signals were visualized and digital images were obtained using a Zeiss LSM 510 confocal microscope equipped with argon and helium-neon lasers (Carl Zeiss, Germany) or using a Zeiss ApoTome microscope coupled to a monochrome charge-coupled device camera (AxioVision, Carl Zeiss). Morphometric measurements of lymphatic and blood vessels in tumor and SLN were made from immunostained tissue sections by photographic analysis using ImageJ software (http://rsb.info.nih.gov/ij) after converting the images into 8-bit gray scale. Measurements of the number and size of the lymphatic vessels having lumen in tumor section were made at a screen magnification of 100×, each 0.84 mm^2 ^in area. Measurements of the densities of the blood vessels in tumor section were made at a screen magnification of 100×, each 0.84 mm^2 ^in area, whereas those and pan-cytokeratin stained cancer cells in SLN were made in total sectioned area (0.3-1.2 mm^2^), and 4 to 5 mice were used per group. To exclude background fluorescence, only pixels over a certain level (>50 intensity value) were taken. Measurements of the densities of the lymphatic vessels and blood vessels in the whole mount staining of bladder were made on whole fields at a screen magnification of 25×, relative densities were calculated in each 1.16 mm^2 ^in area, and 4-5 mice were used per group. Values were expressed as relative densities (%). To measure the size of tumor and SLN, the mid-sectioned tissues were stained with H&E. The stained tissues were photographed with a Zeiss microscope, surface areas of mid-sectioned tumor and SLN were calculated using a Zeiss Apotome microscope coupled to monochrome charge-coupled device (CCD) camera and image analysis software (AxioVision, Zeiss).

### Enrichment of CD11b^+ ^cells from OUBC by FACS and RT-PCR

Single cell suspensions of OUBC were made by incubation with collagenase type IV (Worthington Biochemical Corporation, Lakewood, NJ) for 1 hr, and the suspended cells were washed with FACS buffer (Hank's Buffered Salt Solution (HBSS) + 4% bovine serum). The cells were incubated for 15 min with rat monoclonal PE-conjugated anti-mouse CD11b antibody 1:250 (BD Pharmingen). Dead cells were excluded by staining with 7-amino-actinomycin D (7-AAD, Invitrogen, Carlsbad, CA). CD11b^+ ^cells and CD11b^- ^cells were enriched by a FACS Aria II (BD Biosciences) according to the manufacturer's instructions. The purities of subpopulations of the enriched CD11b^+ ^cells and CD11b^- ^were ~98% according to the FACS analysis. Total RNA from the enriched CD11b^+ ^cells and the CD11b^- ^cells and normal bladder were extracted using Trizol (Invitrogen) and Qiagen RNeasy micro kit (Qiagen, Valencia, CA) according to the manufacturer's instructions. Each cDNA was made with Superscript II reverse transcription system (Invitrogen). Quantitative RT-PCR was performed with the Prime Q-Mastermix (Genet bio, Chungnam, Korea) using the CFX96™ Real-time PCR system (Bio-Rad, Hercules, CA). PCR reactions were performed with the appropriate primers (Table 1) for 60 cycles.

**Table 1:** Primers for real-time RT-PCR

Genes	Primer Sequences	
**VEGF-C**	Forward	5'-CGT TCT CTG CCA GCA ACA TTA CCA C-3'
	
	Reverse	5'-CTT GTT GGG TCC ACA GAC ATC ATG G-3'

**VEGF-D**	Forward	5'-GTC TGT AAA GCA CCA TGT CCG GGA G-3'
	
	Reverse	5'-CCA CAG GCT GGC TTT CTA CTT GCA C-3'

**HPRT**	Forward	5'-CCT CAT GGA CTG ATT ATG GAC A-3'
	
	Reverse	5'-ATG TAA TCC AGC AGG TCA GCA A-3'

### Administration of blocking or depletion agent

To block VEGF-C/D, the mice were treated with tail vein injections of 1.0 × 10^9 ^plaque-forming units (pfu) of adenovirus encoding soluble VEGFR-3 (Ade-sVR3) [25,26] twice on the day and two weeks after the MBT-2 implantation. For systemic depletion of CD11b^+^/CD68^+ ^TAM, the mice were treated with intraperitoneal injections of clodronate liposome (CDL, 25 mg/kg every 3 days i.p.) as previously described at 2 weeks after the tumor implantation [22]. As a negative control, empty control liposome (CL) was injected in the same manner.

### Statistical analysis

Values presented are means ± standard deviation (SD). Significant differences between means were determined by Student's *t*-test or analysis of variance followed by the Student-Newman-Keuls test. The accepted level of statistical significance was p < 0.05.

## Results

### OUBC presents profound lymphangiogenesis, angiogenesis and TAM infiltration in tumor mass

**Figure 1:**
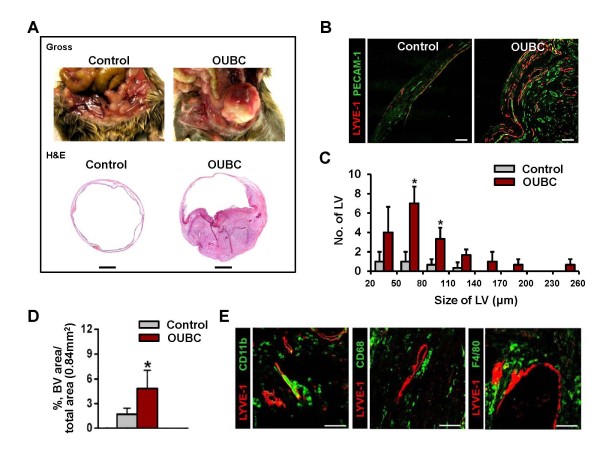
**OUBC presents profound lymphangiogenesis, angiogenesis and CD11b**^+^**/CD68**^+ ^**TAM infiltration**. PBS (Control) or 1 × 10^6 ^MBT-2 cells (OUBC) were injected into the urinary bladders of 8-10-week old female C3H mice. 4 weeks after PBS or the tumor cell injection, the bladders were harvested and stained. (**A**) Photos and images showing gross features and sections (H&E stained) of bladders. Scale bars, 2 mm. (**B**) Images showing LYVE-1^+ ^lymphatic vessels (LV) and PECAM-1^+ ^blood vessels (BV). Scale bars, 100 μm. (**C**) Comparison of LV numbers at different sizes of LV in the bladder sections. Graph shows mean ± SD; n = 5 for each group. *p < 0.05 versus Control. (**D**) Comparison of blood vessel densities (BVD) per total sectioned area (0.84 mm^2^) of bladder. Graph shows mean ± SD; n = 5 for each group. *p < 0.05 versus Control. (**E**) Images showing CD11b^+^, CD68^+ ^and F4/80^+ ^TAM infiltrated near LYVE-1^+ ^lymphatic vessels in OUBC. Scale bars, 100 μm.

Four weeks after the intravesical injection of MBT-2 cells, the lumen of urinary bladder was filled 70-80% with the primary tumor mass (n = 12) (Figure 1A upper). In H&E staining, the tumor invaded the mucosa, submucosa, and muscle layer of bladder wall, which means that OUBC is an invasive bladder tumor model, and not a superficial bladder tumor (Figure 1A lower, Additional File 1). Lymphatic and blood vessels were identified by immunofluorescence staining for LYVE-1 (specific marker for lymphatic endothelial cells) and PECAM-1 (specific marker for blood endothelial cells) in sectioned samples of OUBC and control mice. Lymphatic and blood vessels in OUBC were robust and tortuous, and were densely compacted (Figure 1B). The overall number and size of the lymphatic vessels in the bladder wall were increased in OUBC compared to the control (Figure 1C), and the density of those vessels was ~3.0-fold higher in OUBC as well (Figure 1D). CD11b^+^, CD68^+ ^or F4/80^+ ^TAM was largely infiltrated around lymphatic vessels of tumor mass (Figure 1E). In the CD11b^+ ^TAM, 93 ± 5% (n = 5) was CD68^+ ^and 72 ± 6% (n = 5) was F4/80^+ ^cell (Additional File 2). Thus, TAM adjacent to lymphatic vessels in OUBC was mainly CD11b^+^/CD68^+^/F4/80^+^/LYVE-1^- ^cell.

### OUBC displays marked lymphatic enlargement and increased number of blood vessels in the peritoneal surface of bladder wall

**Figure 2:**
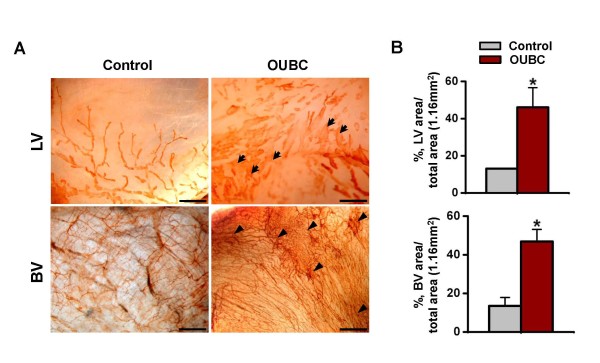
**OUBC displays markedly increased number of lymphatic and blood vessels in the peritoneal surface of bladder wall**. PBS (Control) or 1 × 10^6 ^MBT-2 cells (OUBC) were injected into the urinary bladders of 8-10-week old female C3H mice. 4 weeks after PBS or the tumor cell injection, bladder was whole-mounted and immunostained for LYVE-1 and PECAM-1. (**A**) LYVE-1^+ ^LVs and PECAM-1^+ ^BVs in bladder surface were visualized with DAB staining (brown). Scale bars, 200 μm. Arrows indicate profound and tortuous lymphangiogenesis and arrowheads indicate robust increase of BV networks. (**B**) Comparison of lymphatic vessel densities (LVD) and BVD per total area (1.16 mm^2^) of bladder. Graph shows mean ± SD; n = 5 for each group.*p < 0.05 versus Control.

To examine changes in lymphatic and blood vessels in the peritoneal surface of bladder, the samples were whole mounted and immunostained for LYVE-1 or PECAM-1. Compared to the control, lymphatic vessels on the peritoneal surface were largely and variably enlarged, and the number of blood vessels was higher in the OUBC (Figure 2A). The densities of lymphatic and blood vessels were 2.6- and 3.5-fold higher, respectively in the OUBC (n = 5) than the control (n = 4) (Figure 2B).

### OUBC showed lymphatic metastasis of cancer cells to medial iliac lymph node

**Figure 3:**
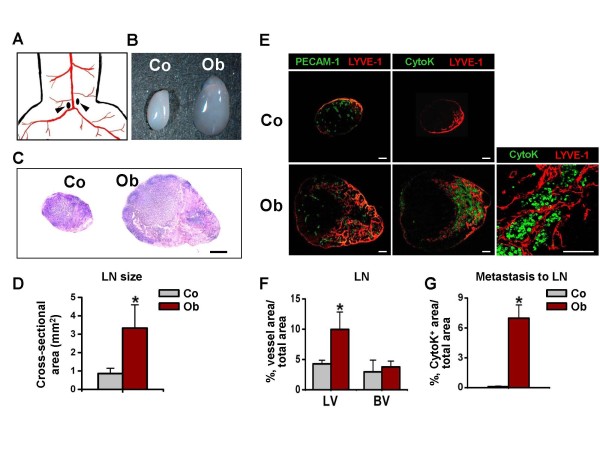
**OUBC displays profound increases in sizes of SLN and metastatic spread of cancer cells to SLN**. PBS (Control, Co) or 1 × 10^6 ^MBT-2 cells (OUBC, Ob) were injected into the urinary bladders of 8-10-week old female C3H mice. 4 weeks after PBS or tumor cell injection, medial iliac lymph nodes (LN) (*arrows*, **A**) were harvested as SLN, and photographed (**B**), and H&E stained (**C**, scale bars, 100 μm). (**D**) Comparison of cross-sectional areas of LNs. Graph shows mean ± SD; n = 5 for each group. *p < 0.05 versus Co. (**E**) Images showing LYVE-1^+ ^LVs, PECAM-1^+ ^BVs, and cytokeratin^+ ^(CytoK^+^) metastatic cancer cells in LNs. Right panel; high magnification view showing that the CytoK^+ ^metastatic cancer cells congregate closely to the lymph node lymphatic vessels of OUBC. Scale bars, 50 μm. (**F**) Comparison of LVD and BVD per total sectioned area of LNs. Graph shows mean ± SD; n = 5 for each group. *p < 0.05 versus Co. (**G**) Comparison of densities of CytoK^+ ^metastatic cancer cells in LNs. Graph shows mean ± SD; n = 5 for each group. *p < 0.05 versus Co.

To examine the lymphatic spread of OUBC-derived cancer cells to the lymph node, we harvested the medial iliac lymph node that lies just above the aortic bifurcation site (Figure 3A) as SLN at 4 weeks after the MBT-2 cell injection. In the OUBC, sizes of SLNs were substantially larger (~3.8 fold) than those of control mice, and the structures were significantly disorganized (Figure 3B, C, and 3D). The densities of the lymphatic vessels in the medial iliac lymph nodes in the OUBC were ~2.3 fold higher than those of the control, whereas the densities of the blood vessels in SLN did not differ significantly from those of the control mice (Figure 3E left and 3F). To search for metastatic tumor cells out in SLN, sections of the lymph node were immunofluorescence stained with pan-cytokeratin, which is a useful marker for detecting of majority of epithelial-origin carcinomas [27,28]. There were many pan-cytokeratin stained cells in the medial iliac lymph nodes of all OUBC mice (Density: 7.0 ± 1.3%, n = 5), while there were no pan-cytokeratin stained cells in the lymph node of control mice (n = 4) (Figure 3E right and 3G). Higher magnification image revealed that metastatic tumor cells congregated closely around lymphatic vessels in the SLN (Figure 3E lower right), indicating that the OUBC induces profound lymphatic metastasis of tumor cells from the primary tumor mass to the SLN.

### VEGFR-3 expressing CD11b^+ ^TAM is the source of VEGF-C/D

**Figure 4:**
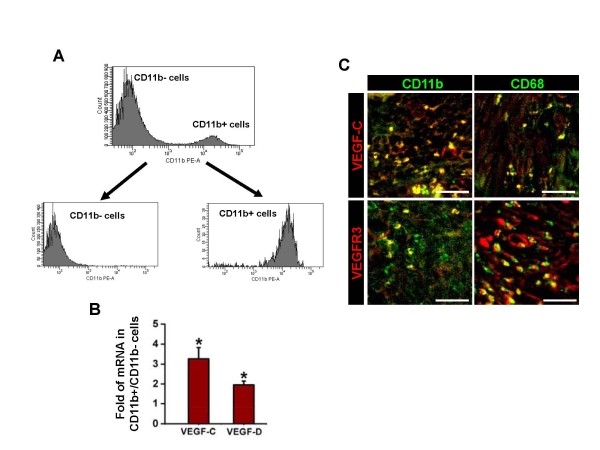
**CD11b**^**+ **^**or CD68**^**+ **^**TAM abundantly express VEGF-C/D and VEGFR-3**. 1 × 10^6 ^MBT-2 cells were injected into the urinary bladders of 8-10-week old female C3H mice. 4 weeks after the tumor cell injection, single cell suspension of OUBC was prepared. (**A**) CD11b^+ ^cells and CD11b^- ^cells were enriched by FACS with ~95% purity. (**B**) Comparison of mRNA expressions of VEGF-C/D in CD11b^+ ^cells. Data are presented as relative fold to CD11b^- ^cells after standardization with hypoxanthine-guanine phosphoribosyl transferase (HPRT). Graph shows mean ± SD; n = 3 for each group. *p < 0.05 versus CD11b^- ^cells. (**C**) Images showing CD11b^+ ^or CD68^+ ^TAM and VEGF-C^+ ^or VEGFR-3^+ ^cells in the sectioned samples. Scale bars, 50 μm.

The result of quantitative reverse transcription-polymerase chain reaction (qRT-PCR) assay revealed that expression levels of VEGF-C/D in bladder tumor mass are much higher than those of normal bladder by 258.3 times and 321.0 times, respectively. To investigate the source and expression of VEGF-C/D that drive OUBC-induced lymphangiogenesis, we performed a series of qRT-PCR assays in the CD11b^+ ^TAM enriched by fluorescent activated cell sorter (FACS) from OUBC (Figure 4A). qRT-PCR analysis revealed that enriched CD11b^+ ^TAM expressed higher amount of VEGF-C (3.3-fold) and VEGF-D (2.0-fold) when compared to the CD11b^- ^cells in bladder tumor mass (Figure 4B). Co-immunostaining analysis showed that CD11b^+ ^or CD68^+ ^TAM expressed VEGF-C (Figure 4C upper). Moreover, VEGFR-3 was highly expressed not only in the lymphatic vessels but also in the CD11b^+ ^or CD68^+ ^TAM of the OUBC (Figure 4C lower).

### Blockade of VEGF-C/D by soluble extracellular domain of VEGFR-3 suppressed OUBC-induced lymphangiogenesis and lymphatic metastasis to SLN but not CD11b^+ ^TAM recruitment

**Figure 5:**
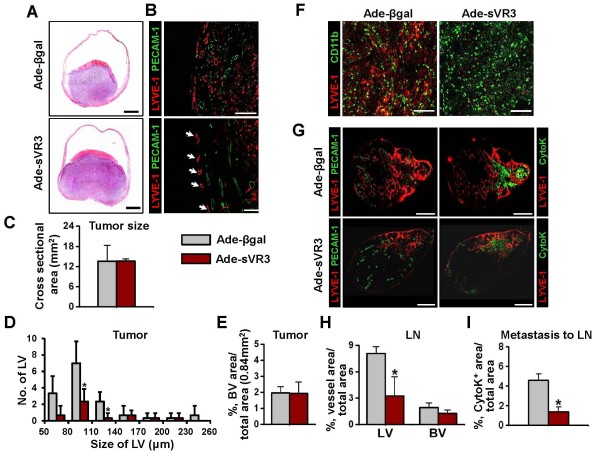
**Blockade of VEGF-C/D-VEGFR-3 signaling suppresses profound lymphangiogenesis and lymphatic metastasis, but not recruitment of CD11b**^+^**/CD68**^+ ^**TAM**. 1 × 10^6 ^MBT-2 cells were injected into the urinary bladders of 8-10-week old female C3H mice. At the day of and two weeks after the tumor cell injection, 1 × 10^9 ^pfu Ade-βgal or Ade-sVR3 were injected intravenously to the OUBC mice. 4 weeks after the tumor cell injection, the bladder and the SLN were sampled. (**A**) Images showing H&E stained sections of tumors. Scale bars, 2 mm. (**B**) Comparison of cross-sectional area of tumors. Graph shows mean ± SD; n = 5 for each group. (**C**) Images showing LYVE-1^+ ^LVs and PECAM-1^+ ^BVs in tumors. Scale bars, 200 μm. (**D**) Comparison of LV numbers at different sizes of LV in the tumor sections. Graph shows mean ± SD; n = 5 for each group. *p < 0.05 versus Ade-βgal. (**E**) Comparison of BVD per total sectioned area of tumors. Graph shows mean ± SD; n = 5 for each group. (**F**) Images showing CD11b^+ ^TAM and LYVE-1^+ ^cells. Scale bars, 200 μm. (**G**) Left panel; images showing LYVE-1^+ ^LVs and PECAM-1^+ ^BVs in LNs. Right panel; images showing LYVE-1^+ ^LVs and CytoK^+ ^metastatic cancer cells in LNs. Scale bars, 500 μm. (**H**) Comparison of LVD and BVD in LNs. Graph shows mean ± SD; n = 5 for each group. *p < 0.05 versus Ade-βgal. (**I**) Comparison of densities of CytoK^+ ^metastatic cancer cells in LNs. Graph shows mean ± SD; n = 5 for each group. *p < 0.05 versus Ade-βgal.

To investigate the effects of VEGF-C/D in the OUBC, soluble extracellular domain of VEGFR-3 (sVR3) was overexpressed by systemic adenoviral delivery (hereafter referred as 'Ade-sVR3') [29] (n = 5). As a control, same amount of Ade-βgal was treated in the same manner (n = 4). At four weeks after the cancer cell implantation, the number of the lymphatic vessels in tumor treated with Ade-sVR3 showed significant decrease of 65% over that of Ade-βgal-treated tumor (Figure 5B and 5D). However, there were no significant changes in the growth of tumor sizes and the densities of blood vessels between the Ade-sVR3 versus the Ade-βgal (Figure 5A,B,C and 5E). It was noted that blockade of VEGF-C/D-VEGFR3 signaling is effective in disrupting lymphangiogenesis in bladder tumor. Furthermore, distribution densities of CD11b^+ ^TAM in OUBC were indistinguishable between Ade-sVR3 and Ade-βgal, suggesting that VEGF-C/D signaling has no effect on the recruitment of CD11b^+ ^TAM into the OUBC (Figure 5F). In SLN, the Ade-sVR3 significantly decreased densities of lymphatic vessels, but not blood vessels (Figure 5G and 5H). Importantly, metastatic cancer cells in SLN were decreased by 60% in the Ade-sVR3 compared to the Ade-βgal (Figure 5I), indicating that blockade of VEGF-C/D by Ad-sVR3 strongly suppressed lymphatic metastasis to SLN.

### CD11b^+ ^TAM plays a crucial role in OUBC-induced lymphangiogenesis

**Figure 6:**
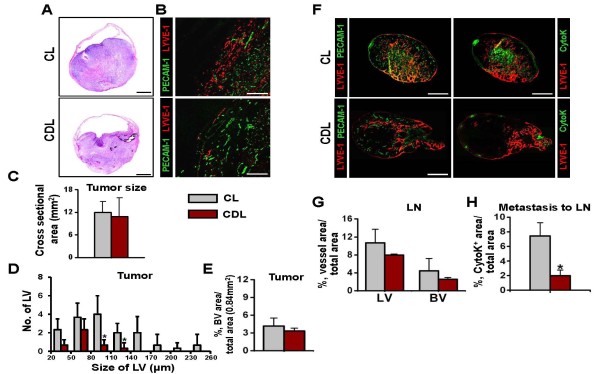
**Depletion of CD11b**^**+**^**/CD68**^**+ **^**TAM reduced OUBC-induced lymphangiogenesis and lymphatic spread of bladder cancer cells**. 1 × 10^6 ^MBT-2 cells were injected into the urinary bladders of 8-10-week old female C3H mice. 2 weeks after the tumor cell injection, clodronate liposome (CDL) or control liposome (CL) were injected intraperitonealy to the OUBC mice once every 3 days. 4 weeks after the tumor cell injection, the bladder and SLN were sampled. (**A**) Images showing H&E stained sections of tumors. Scale bars, 2 mm. (**B**) Comparison of cross-sectional area of tumors. Graph shows mean ± SD; n = 5 for each group. (**C**) Images showing LYVE-1^+ ^LVs and PECAM-1^+ ^BVs in tumors. Scale bars, 200 μm. (**D**) Comparison of LV numbers at different sizes of LV in the tumor sections. Graph shows mean ± SD; n = 5 for each group. *p < 0.05 versus CL. (**E**) Comparision of BVD per total sectioned area of tumors. Graph shows mean ± SD; n = 5 for each group. (F) Left panel; images showing LYVE-1^+ ^LVs and PECAM-1^+ ^BVs in LNs. Right panel; images showing LYVE-1^+ ^LVs and CytoK^+ ^metastatic cancer cells in LNs. Scale bars, 500 μm. (G) Comparison of LVD and BVD in LNs. Graph shows mean ± SD; n = 5 for each group. (**I**) Comparison of densities of CytoK^+ ^metastatic cancer cells in LNs. Graph shows mean ± SD; n = 5 for each group. *p < 0.05 versus CL.

To clarify the role of infiltrating CD11b^+ ^TAM in the OUBC-induced lymphangiogenesis and angiogenesis, we depleted macrophages, including CD11b^+ ^TAM, systemically by using the treatment of clodronate liposome (CDL) [30,31] (n = 5). As a control, control liposome (CL) was treated in the same manner (n = 4). Two weeks after the CDL treatment, more than 90% of CD11b^**+**^/CD68^**+ **^TAM were depleted in the tumor region of the OUBC, whereas the CL treatment did not affect the number of CD11b^+^/CD68^+ ^TAM in the tumor (Additional File 3). Although tumor sizes were indistinguishable between CDL treatment versus the CL treatment (Figure 6A and 6C), the CDL treatment decreased the number of lymphatic vessels in bladder tumor by 74% (Figure 6B and 6D). The CDL treatment also showed the tendency to decrease blood vessel densities, but it was not statistically significant (Figure 6B and 6E). These data suggest that CD11b^+ ^TAM plays a critical role in inducing lymphangiogenesis in the OUBC.

### Lymphatic metastasis of the OUBC was strongly inhibited by depletion of CD11b^+ ^TAM

To elucidate the role of CD11b^**+ **^TAM in lymphatic metastasis in the OUBC, the SLN of CDL and CL treated mice were examined. Treatment with CDL, but not CL, reduced the growth of lymphatic and blood vessels in the SLN (Figure 6F left). The densities of lymphatic and blood vessels in the SLN were decreased by about 30% and 40% in the CDL treatment compared to the CL treatment, respectively (Figure 6G). The lymphatic metastasis to the SLN was remarkably decreased by 70% in the CDL treated group when compared to the CL treated control group (Figure 6F right and 6H), indicating that CD11b^+ ^TAM play crucial role in lymphatic metastasis of bladder cancer cells.

## Discussion

To establish distant metastasis, bladder tumor cells spread from the bladder to target organs through three main routes: hematogenous spread, lymphatic spread, and direct seeding. In bladder cancer, lymph node was known as the most common site of metastasis. Nearly 70% of bladder cancer patients experience lymphatic metastasis in the course of disease progression [2]; thus, lymph node metastasis is an important initial step toward distant metastasis [32].

In present study, OUBC model was an invasive bladder cancer which invades all layers of bladder wall and displayed profound lymphangiogenesis in primary tumor mass and SLN. Because TAM is known to play an important role in tumor lymphangiogenesis by secreting lymphangiogenic growth factor in other type of cancers [20-22], we proposed that profound lymphangiogenesis is associated with TAM, and bladder cancer cells can actively metastasize through lymphatic vessels with the help of TAM. In fact, one previous report indicates that TAM is frequently observed in clinical samples of bladder cancer [33], and that patients with high TAM count showed higher frequencies of vascular invasion and distant metastasis, and have lower 5-year survival rates than those with low TAM count. Moreover, the TAM count and microvessel densities were positively correlated to each other. Indeed, the present study showed that there are massive infiltration of CD11b^+ ^macrophages located near the lymphatic vessels in primary tumor and SLN. Moreover, immunofluorescence staining for the markers such as CD68 and F4/80 revealed that most of TAM is the tumor and metastasis-promoting M2-type [17].

The depletion of TAM by using CDL dramatically decreased the lymphangiogenesis in tumor and SLN and suppressed lymphatic metastasis of tumor cells to SLN, indicating that TAM is an important pro-lymphangiogenic and pro-metastatic modulator in the bladder tumor. In addition to increasing the number of lymphatic vessels, TAM is able to condition the vessels to be vulnerable to intravasation of tumor cells: TAM facilitates migration and intravasation of tumor cells by secreting growth factors and chemotactic factors, and by remodeling the extracellular matrix through collagen fibril formation [34]. Therefore, in the present study, it was possible that the anti-metastatic effect of CDL treatment might have resulted from not only the suppression of TAM-induced angiogenesis and lymphangiogenesis, but also from the inhibition of TAM-induced tumor cell migration and intravasation. However, contrary to previous report [31], depletion of TAM by CDL did not significantly reduce the tumor mass in the bladder. This could have resulted from the different characteristics of tumors in growing different organs. This orthotopic tumor grows mainly into the bladder cavity without invading its supporting tissues, a growth pattern that is quite different from that of other solid tumors.

Interestingly, CD11b^+^/CD68^+ ^TAM expresses higher levels of VEGF-C/D than CD11b- cells, which were also known as the main mediator of tumor-associated lymphangiogenesis and lymphatic metastasis. Because CD11b^+^/CD68^+ ^TAM flocked near the lymphatic vessels and VEGFR-3 was highly expressed in the lymphatic vascular endothelial cells, TAM could assist tumor-induced lymphangiogenesis by paracrine secretion of VEGF-C/D in bladder cancer. Moreover, co-immunostaining results showed that VEGFR-3 is also highly expressed in the CD11b^+ ^TAM in itself, indicating that TAM may have autocrine and paracrine positive feedback loop to boost further secretion of VEGF-C/D. Consequently, once TAM is activated to become M2-type, it could accelerate lymphangiogenesis and lymphatic metastasis by the paracrine and autocrine mechanism. From this study, we could not exclude the partial contribution of tumor cells to the source of VEGF-C/D. Nevertheless, we were able to exclude the idea that tumor cells are the main source of tumor lymphangiogenesis, because depletion of macrophages by the CDL treatment caused >80% reduction in VEGF-C/D expressions in the tumor mass compared to the control CL treatment.

In addition, present data also indicate that the suppression of VEGF-C/D signaling by adenoviral delivery of sVR3 significantly reduced the growth of lymphatic vessels in bladder tumor and SLN and eventually inhibited lymphatic metastasis of bladder tumor cells from primary tumor to SLN without significant reductions of tumor mass and blood vessels in OUBC. These findings are mostly consistent with previously reported findings in the different tumor models [11-13]. Therefore, we postulated that lymphatic spread of cancer metastasis in patients with bladder cancer can be prevented by specific suppression of VEGF-C/D-VEGFR3 signaling pathways. But, this strategy seemed to have a limitation when reducing preformed tumor burden. In other words, because blocking lymphangiogenesis is rather tumoristatic than tumoricidal, blocking VEGF-C/D might be particularly useful when tumor burden is minimal, for example in the postoperative adjuvant setting. In comparison, recent studies [35,36] show that VEGFR-3 is upregulated in the tumor vasculature; also, loss-of-function studies by genetic targeting of VEGFR-3 or blocking of its signaling with monoclonal antibodies resulted in decreased sprouting, indicating that VEGFR-3 has a role in angiogenic sprouting formation. The discrepancies in responsiveness to VEGFR-3 blocking experiments could have resulted from the different expression level of VEGFR-3 in the tumor vasculatures in varying tumor models. Indeed, the vasculatures and the sizes of our bladder cancer models are not responsive to the soluble VEGFR-3 treatment, which might be due to the low expression of VEGFR-3 in the tumor vasculatures.

Until now, even after successful radical cystectomy and lymph node dissection, about 50% of bladder cancer patients eventually show recurrence and have poor survival [1]. For more than 30 years, there have been many efforts to reduce the recurrence of high-risk bladder cancer after curative surgery, however, numerous clinical trials failed to show conclusive evidence on the impact of adjuvant chemotherapy even by using the most toxic combination regimens [37]. Considering these facts, blocking VEGF-C/D pathway and lymphangiogenesis could be another attractive alternative to reduce the recurrence of bladder tumor after curative radical cystectomy.

## Conclusion

In summary, a profound OUBC-induced lymphangiogenesis in primary cancer and SLN contribute to lymphatic metastasis by VEGF-C/D and VEGFR-3 signaling. Infiltrated CD11b^+^/CD68^+ ^TAM is intensely involved in OUBC-induced lymphangiogenesis and lymphatic metastasis and therefore TAM could be the bridge between primary cancer and SLN. Our study, which shows the therapeutic inhibition of lymphangiogenesis, provides another therapeutic target to inhibit lymphatic metastasis and recurrence in patients with invasive bladder cancer.

## Competing interests

The authors declare that they have no competing interests.

## Authors' contributions

HY, CK, WJK, KA and GYK designed and organized the experiments. WH provided MBT-2 cell line. HY, CK, MJK, and GYK performed the animal studies, analyzed the data, generated the figures, and histological analyses. RAS prepared materials for the experiments. HY, CK, WJK, WH and GYK wrote the manuscript. All authors read and approved the final manuscript.

## Supplementary Material

Additional file 1 **OUBC is a muscle-invasive bladder cancer**. PBS (Control) or 1 × 10^6 ^of MBT-2 cells (OUBC) were injected into the urinary bladder of 8-10-week old female C3H mice. 4 weeks after PBS or tumor cell injection, the bladders were harvested and stained with H&E. Images showing sectioned bladders. Arrows indicate the tumor cells which invaded the muscle layer. Click here for file

Additional file 2 **Most TAM in OUBC are CD11b**^**+**^**/CD68**^**+**^**/F4/80**^**+ **^**cells**. 1 × 10^6 ^of MBT-2 cells were injected into the urinary bladder of 8-10-week old female C3H mice. 4 weeks after tumor cell injection, the bladders were harvested and stained. Images showing CD11b^+^, CD68^+ ^and F4/80^+ ^TAM in tumors. Scale bars, 100 μm. Click here for file

Additional file 3 **Depletion of TAM by CDL in OUBC**. 1 × 10^6 ^of MBT-2 cells were injected into the urinary bladder of 8-10 week old female C3H mice. 2 weeks after tumor cell injection, CDL or CL was intraperitonealy injected to the mice bearing OUBC at every 3 day. 4 weeks after tumor cell injection, the bladders were harvested and immunostained. (A) Images showing CD11b^+^/CD68^+ ^TAM in the CL or CDL treated mice. Scale bars, 100 μm. (B) High magnification view showing the depletion of CD11b^+ ^TAM in CDL treated mice compared to CL treated mice. Scale bars, 100 μm. Click here for file
