# Supplementary figures and images for: Soluble vascular endothelial growth factor receptor-3 suppresses lymphangiogenesis and lymphatic metastasis in bladder cancer
Source: Mol Cancer. 2011 Apr 11;10:36. doi: 10.1186/1476-4598-10-36 (PMC3080348; doi:10.1186/1476-4598-10-36)

**Control**

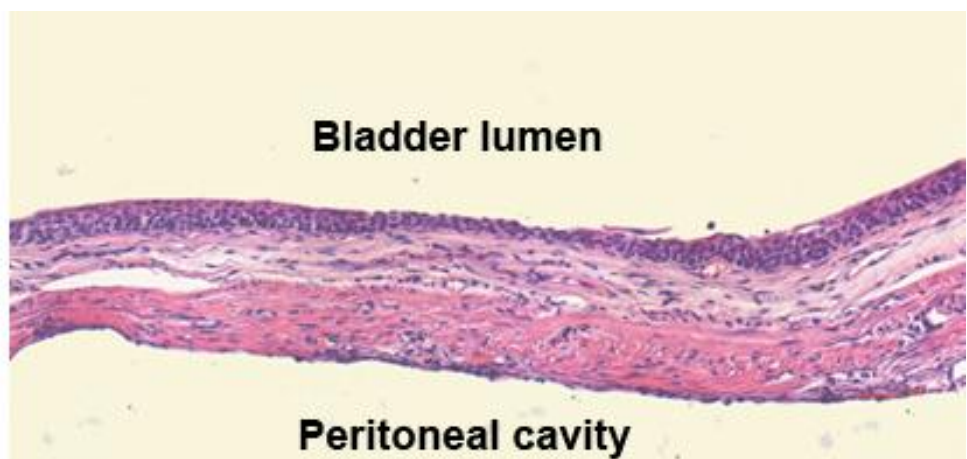

**OUBC**

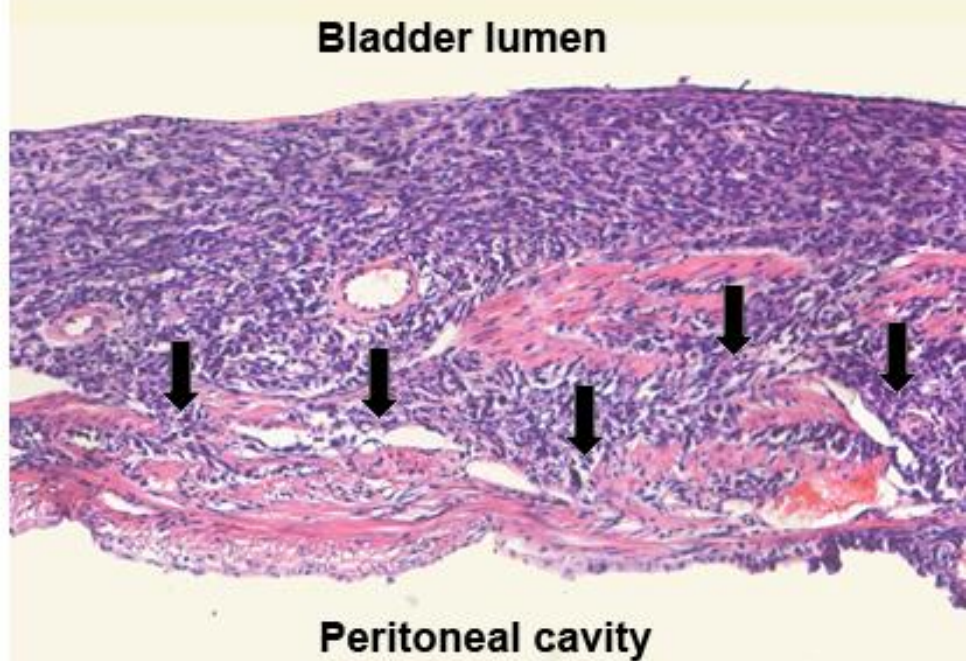

Supplement: Additional file 1 — OUBC is a muscle-invasive bladder cancer. PBS (Control) or 1 × 10^6^ of MBT-2 cells (OUBC) were injected into the urinary bladder of 8-10-week old female C3H mice. 4 weeks after PBS or tumor cell injection, the bladders were harvested and stained with H&E. Images showing sectioned bladders. Arrows indicate the tumor cells which invaded the muscle layer. [file 1476-4598-10-36-S1.PDF]

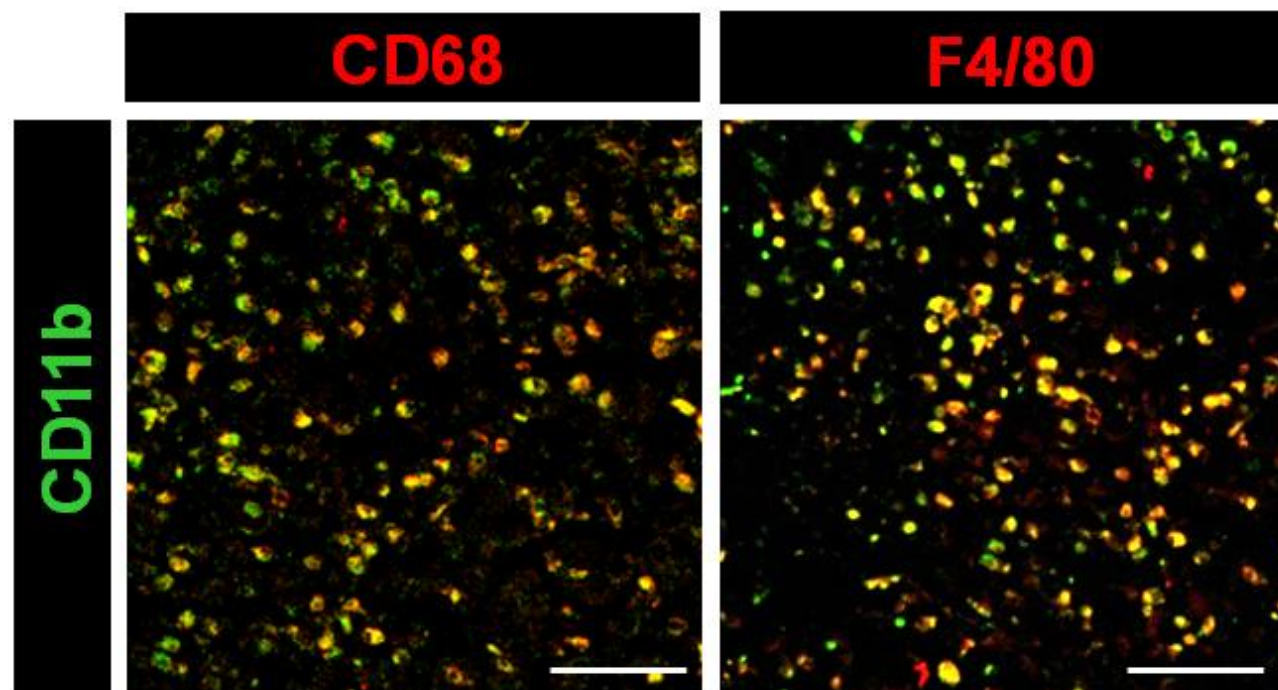

Supplement: Additional file 2 — Most TAM in OUBC are CD11b+/CD68+/F4/80+ cells. 1 × 10^6^ of MBT-2 cells were injected into the urinary bladder of 8-10-week old female C3H mice. 4 weeks after tumor cell injection, the bladders were harvested and stained. Images showing CD11b+, CD68+ and F4/80+ TAM in tumors. Scale bars, 100 μm. [file 1476-4598-10-36-S2.PDF]
