# Supplementary material for: Soluble vascular endothelial growth factor receptor-3 suppresses lymphangiogenesis and lymphatic metastasis in bladder cancer
Source: Mol Cancer. 2011 Apr 11;10:36. doi: 10.1186/1476-4598-10-36 (PMC3080348; doi:10.1186/1476-4598-10-36)
Supplement: Additional file 3 — Depletion of TAM by CDL in OUBC. 1 × 10^6^ of MBT-2 cells were injected into the urinary bladder of 8-10 week old female C3H mice. 2 weeks after tumor cell injection, CDL or CL was intraperitonealy injected to the mice bearing OUBC at every 3 day. 4 weeks after tumor cell injection, the bladders were harvested and immunostained. (A) Images showing CD11b+/CD68+ TAM in the CL or CDL treated mice. Scale bars, 100 μm. (B) High magnification view showing the depletion of CD11b+ TAM in CDL treated mice compared to CL treated mice. Scale bars, 100 μm. [file 1476-4598-10-36-S3.PDF]

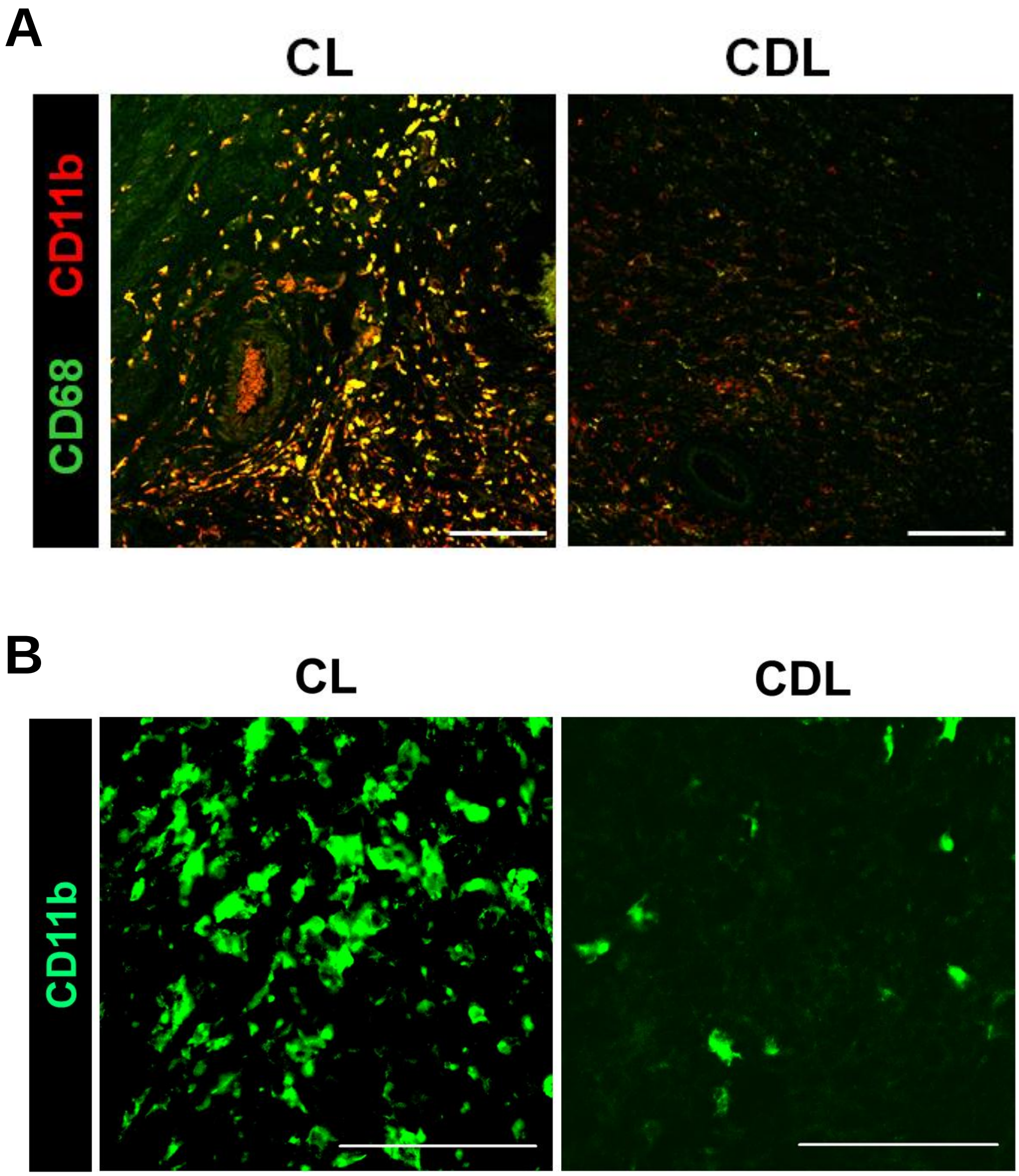

Additional Figure 3. Depletion of TAM by CDL in OUBC

$1x10^6$ of MBT-2 cells were injected into the urinary bladder of 8-10 week old female C3H mice. 2 weeks after tumor cell injection, CDL or CL was intraperitonealy injected to the mice bearing OUBC at every 3 day. 4 weeks after tumor cell injection, the bladders were harvested and immunostained. (**A**) Images showing $CD11b^+/CD68^+$ TAM in the CL or CDL treated mice. Scale bars, 100 μm. (**B**) High magnification view showing the depletion of $CD11b^+$ TAM in CDL treated mice compared to CL treated mice. Scale bars, 100 μm.
